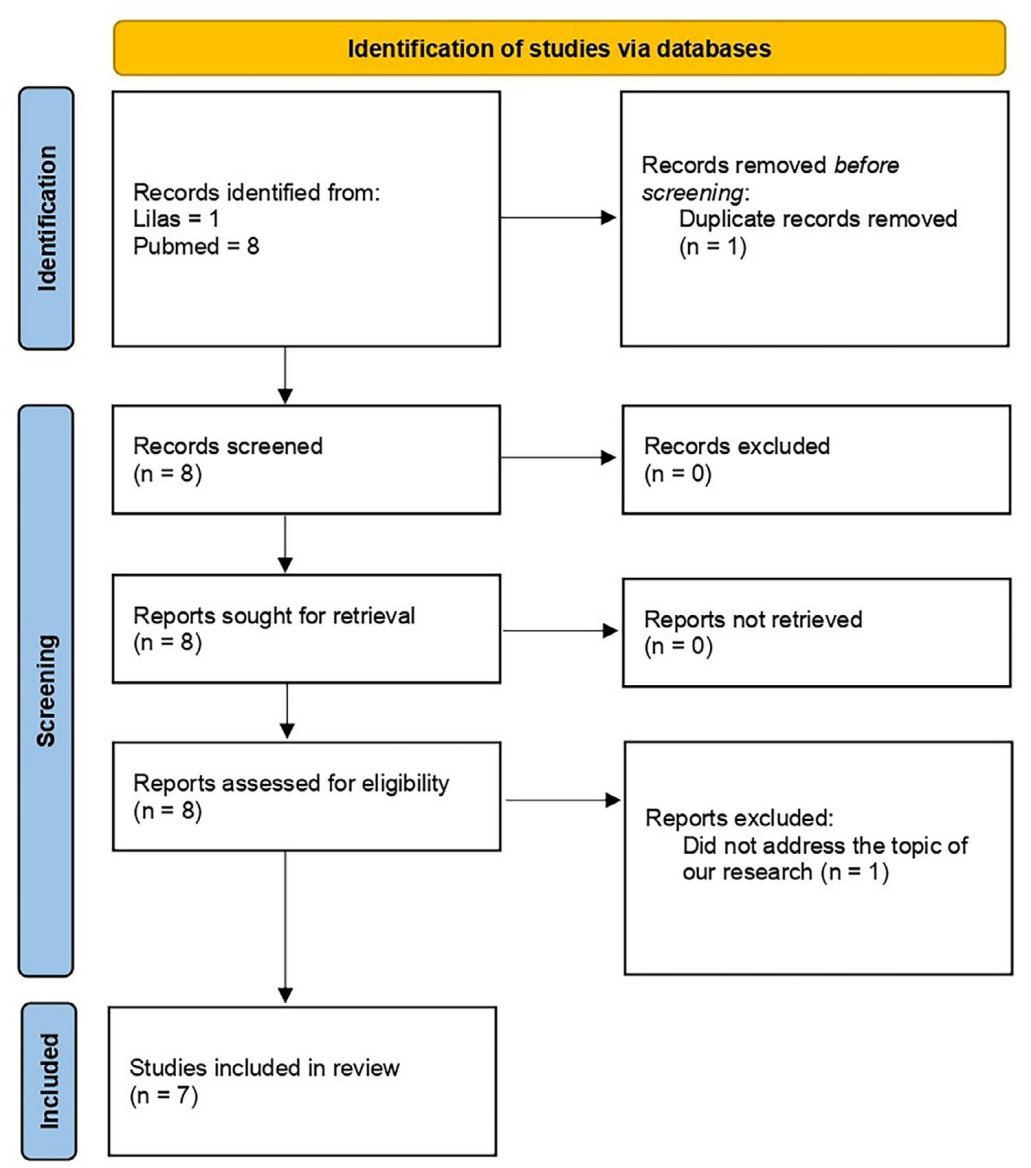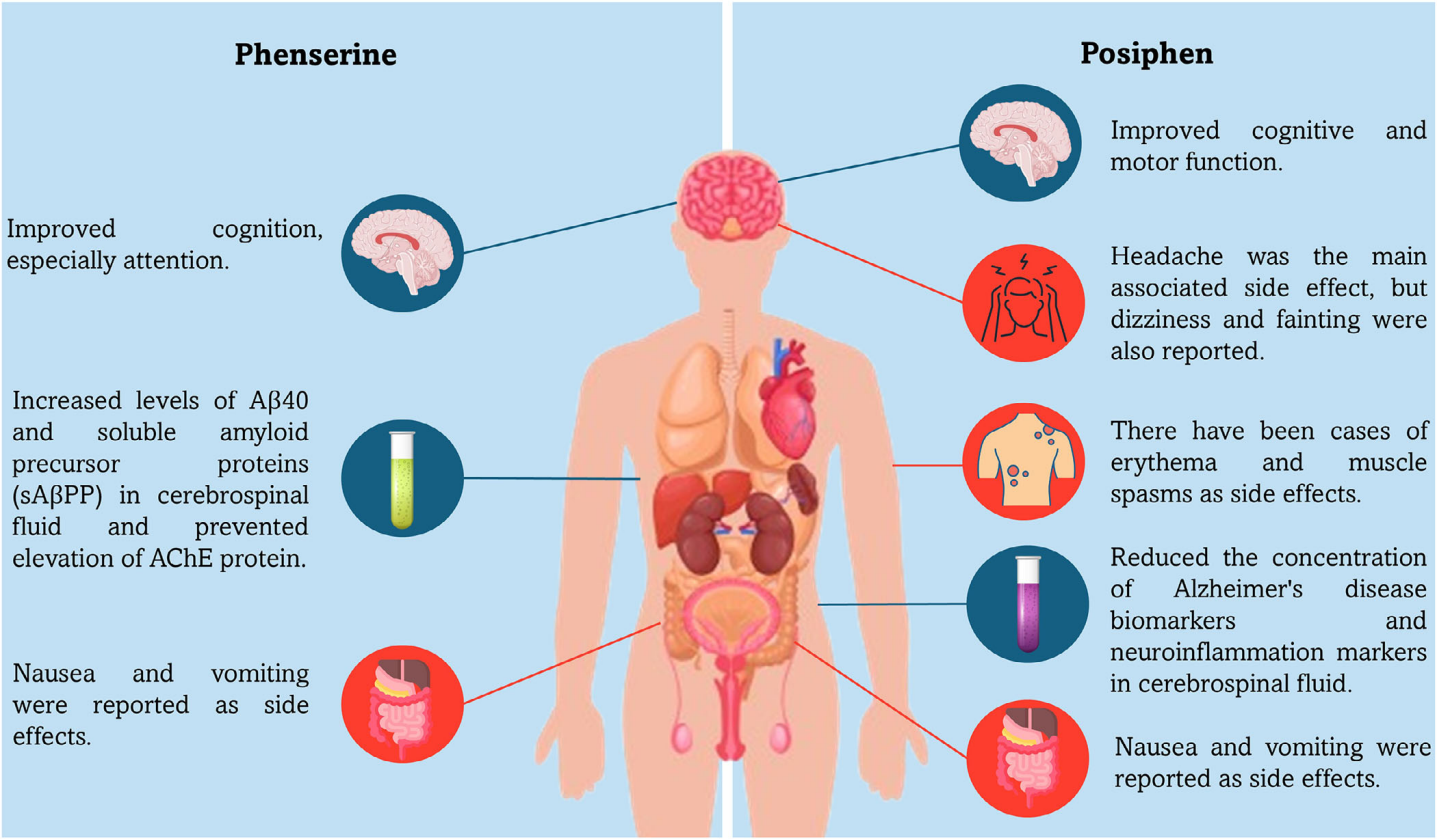# Efficacy and Safety of Posiphen and its Precursor Phenserine in Alzheimer’sDisease: a Systematic Review

**DOI:** 10.1002/alz70859_103854

**Published:** 2025-12-25

**Authors:** Gabriela Oliveira do Nascimento, Kevin Gustavo dos Santos Silva, Pedro Augusto Rodrigues Vinhas, Carlos Rocha Oliveira

**Affiliations:** ^1^ Anhembi Morumbi University, São José dos Campos, São Paulo Brazil; ^2^ Federal University of São Paulo, São José dos Campos, São Paulo Brazil

## Abstract

**Background:**

Phenserine, an acetylcholinesterase inhibitor, increases brain acetylcholine levels and may improve cognitive function, while its positive enantiomer, Posiphen, does not inhibit acetylcholinesterase but reduces beta‐amyloid production by inhibiting APP translation through the 5′ untranslated region (5′UTR) of alpha‐synuclein mRNA, a protein linked to neurodegenerative diseases like Parkinson’s. This study aims to systematically review the efficacy and safety of Posiphen and phenserine in the treatment of Alzheimer’s disease.

**Method:**

This systematic review evaluated the efficacy and safety of Posiphen and Phenserine in Alzheimer's disease treatment. Searches were conducted in Medline (PubMed) and Lilacs/VHL using the terms "posiphen", "buntanetap" or "ANVS401" combined with “Alzheimer Disease” via the Boolean operator “AND,” applying filters for observational studies and clinical trials (randomized or not) published in English or Spanish within the last 15 years. Studies involving animal models, lacking quantitative outcome data, or focused on cognitive impairment from other pathologies were excluded. The risk of bias was assessed using the Revised Cochrane Risk‐of‐bias Tool for Randomized Trials (RoB 2.0).

**Result:**

Seven eligible studies (*n* = 7) were included (Figure 1), of which only the study did not demonstrate cognitive improvement in patients using Posiphen. As illustrated in Figure 2, the studies by Fang et al. (2023) and Maccecchini et al. (2012) showed cognitive improvement, which is associated with reduced concentrations of Alzheimer's disease biomarkers (amyloid precursor proteins sAPPα and sAPPβ), total Tau protein levels, markers of neuroinflammation such as Complement 3 (C3) and YKL4023. Phenserine has also been shown to improve cognition, especially attention. Its adverse effects were limited to nausea and vomiting, while adverse effects of Posiphen included headache, dizziness, fainting, erythema, muscle spasms, nausea, and vomiting.

**Conclusion:**

The reviewed studies suggest that Phenserine and Posiphen may improve cognitive function and reduce Alzheimer's disease biomarkers and markers of neuroinflammation present in cerebrospinal fluid. However, small sample sizes and variability in dosages limit the generalizability of these findings. Future trials with larger cohorts and standardized protocols are needed to validate these results.